# A multi‐institutional study for tolerance and action levels of IMRT dose quality assurance measurements in Korea

**DOI:** 10.1120/jacmp.v14i2.3964

**Published:** 2013-03-04

**Authors:** Jung‐in Kim, Jin‐Beom Chung, Yang‐Kyun Park, Ju‐Young Song, Sung Kyu Kim, Sung Hwan Ahn, Chang Heon Choi, Won Hoon Choi, Byungchul Cho, Sang Gyu Ju, Sung Jin Kim, Sung‐Joon Ye

**Affiliations:** ^1^ Interdisciplinary Program in Radiation Applied Life Science and Institute of Radiation Medicine College of Medicine, Seoul National University Seoul; ^2^ Department of Radiation Oncology KangBuk Samsung Medical Center Seoul; ^3^ Department of Radiation Oncology Seoul National University Bundang Hospital Seongnam; ^4^ Department of Radiation Oncology Seoul National University Hospital Seoul; ^5^ Department of Radiation Oncology Chonnam National University Medical School Hwasun; ^6^ Department of Therapeutic Radiology & Oncology Yeungnam University Seoul; ^7^ Department of Radiation Oncology Dong‐A University Medical Center Busan; ^8^ Department of Radiation Oncology Jeju National University Hospital Jeju; ^9^ Department of Radiation Oncology Yonsei Cancer Center, Yonsei University College of Medicine, Yonsei University Health System Seoul; ^10^ Department of Radiation Oncology Asan Medical Center, University of Ulsan College of Medicine Seoul; ^11^ Department of Radiation Oncology Seoul Samsung Medical Center Seoul; ^12^ Department of Radiation Oncology Eulji University Hospital Daejon; ^13^ Department of Transdisciplinary Studies and Advanced Institutes of Convergence Technology Seoul National University Suwon Korea

**Keywords:** IMRT, confidence limit, tolerance level, dose quality

## Abstract

The purpose of this study was to suggest tolerance levels for IMRT DQA measurements using confidence limits determined by a multi‐institutional study in Korea. Ten institutions were grouped into LINAC (seven linear accelerators) and TOMO (three tomotherapy machines). The DQA processes consisted of point (high‐ and low‐dose regions) and planar (per‐field and composite‐field) dose measurements using an ion chamber and films (or 2D detector array) inserted into a custom‐made acryl phantom (LINAC) or a cheese phantom (TOMO). The five mock structures developed by AAPM TG‐119 were employed, but the prostate as well as the H&N structures were modified according to Korean patients' anatomy. The point measurements were evaluated in a ratio of measured and planned doses, while the planar dose distributions were assessed using two gamma criteria of 2 mm/2% and 3 mm/3%. The confidence limit (|mean + 1.96 σ|) for point measurements was determined to be 3.0% in high‐dose regions and 5.0% in low‐dose regions. The average percentage of points passing the gamma criteria of 2 mm/2% and 3 mm/3% for per‐field measurements was 92.7 ±6.5% and 98.2 ±2.8%, respectively. Thus, the corresponding confidence limit was 79.1% and 92.7%, respectively. The gamma passing rate averaged over all mock tests and institutions for composite‐field measurements was 86.1 ±6.5% at 2 mm/2% and 95.3 ±3.8% at 3 mm/3%, leading to the confidence limit of 73.3% and 87.9%, respectively. There was no significant difference in the tolerance levels of point dose measurements between LINAC and TOMO groups. In spite of the differences in mock structures and dosimetry tools, our tolerance levels were comparable to those of AAPM and ESTRO guidelines.

PACS number: 87.55.Qr

## I. INTRODUCTION

Intensity‐modulated radiation therapy (IMRT) has been rapidly implemented in Korea due to recent governmental policy.^(^ ^1^ ^)^ Roughly three‐fourths of all radiation treatment facilities currently perform IMRT. IMRT is capable of generating extremely precise dose distributions that provide conformal coverage of complex target shapes and conformal avoidance of sensitive normal structures.^(^ ^2^ ^)^ The AAPM (American Association of Physicists in Medicine) and ESTRO (European Society for Therapeutic Radiology and Oncology) emphasized a comprehensive quality assurance program for clinical implementation of IMRT.^(^ ^3^ ^,^ ^4^ ^,^ ^5^ ^)^ Also, the guideline of IMRT use in clinical trials has been developed throughout the world.^(^ ^6^ ^,^ ^7^ ^)^ AAPM Task Group 119 carried out a multi‐institutional research to assess the overall accuracy of planning and delivery of IMRT, and produced quantitative confidence limits as baseline expectation values for IMRT commissioning.^(^ ^8^ ^)^ The British group carried out a national dosimetry audit of IMRT to provide an independent check of safe implementation and to identify problems in the modeling and delivery of IMRT.^(^ ^7^ ^,^ ^9^ ^–^ ^11^ ^)^

With a rapid implementation of IMRT in Korea, ten institutions reached consensus on a multi‐institutional study to provide tolerance levels of IMRT DQA measurements as a national safety guideline for the overall accuracy of IMRT planning and delivery. The confidence limit concept and test protocol of AAPM TG‐119 were used as a basis of this study. However, the mock structures were modified according to Korean patients' anatomy, and the dosimetry tools and phantoms used in this study were somewhat different from those of AAPM TG‐119. Furthermore, ten institutions were grouped into LINAC (seven linear accelerator‐based centers) and TOMO (three tomotherapy‐based centers), and the results of both groups were separately analyzed.

## II. MATERIALS AND METHODS

This study was performed from October 2010 to September 2011. Ten participating institutions geometrically spread over Korea were divided into the LINAC and TOMO groups. The planning and delivery systems used at each institution are summarized in Table 1. Both groups used 6 MV photon beams. The institutions listed in subsequent tables were anonymously identified only by letter.

**Table 1:** List of participating institutions and the planning and delivery systems used.

*Group*				*LINAC*					*TOMO*	
Institution	Seoul Nat'l Univ. Bundang Hosp.	Jeju Nat'l Univ. Hosp	Yeungnam Univ. Hosp.	Dong‐A Univ. Hosp.	Eulji Univ. Hosp.	Seoul Nat'l Univ. Hosp	Asan Medical Center	Seoul Samsung Medical Center	Yonsei Cancer Center	Chonnam Nat'l Univ. Hwasun Hosp.
Accelerator	Varian 21ExS	Varian IX	Varian 21ExS	Varian Novalis	Elekta Synergy	Varian IX	Varian Trilogy	Tomotherapy	Tomotherapy	Tomotherapy
Delivery Technique	DMLC	DMLC	DMLC	DMLC	SMLC	DMLC	DMLC	BMLC	BMLC	BMLC
Planning System	Eclipse 6.5	Eclipse 8.6	Eclipse 8.6	BrainLab iPlan	CMS Monaco 2.0.3	Eclipse 8.9	Eclipse 8.9	Tomotherapy TPS 3.1.4	Tomotherapy TPS 4.0.2	Tomotherapy TPS 3.2.3.2

DMLC=dynamic MLC; SMLC=static MLC; and BMLC=binary MLC.

### A. Output and TPS audit

One of the institutions has been participating in a RTOG (Radiation Therapy Oncology Group) trial and passed the RPC's (Radiological Physics Center. MD Anderson Cancer Center, USA) credentialing requirements. The dose audit was performed with OSLD (Optically Stimulated Luminescence Dosimeter). RPC presented that the variance of the dose determined by a single OSLD was less than 3%. The OSLD chips were irradiated at $d_{\max}$ of photon beams at 100 cm SSD (source‐to‐surface distance) in the institution and then shipped to RPC for analysis. As a result of RPC auditing, the absorbed dose measurements in good agreement were ranged from 0.98 to 1.00. This institution played the role as the reference site to indirectly verify the machine output of the other institutions through site visiting. The output was measured by skilled physicists of the reference site using a farmer type ionization chamber (0.6 cc, PTW TN30013; PTW, Freiburg, Germany) and an electrometer (PTW UNIDOS) calibrated by KRISS (Korea Research Institute of Standards and Science).

The TPS (treatment planning system) commissioning audit was carried out using two test protocols described by Van Esch et al.^(^ ^7^ ^,^ ^12^ ^)^ These tests were designed with three consecutive rectangular volumes that had different specified doses. The first test called a “Dip” test, for which the specified dose to the middle volume and each outer volume was 0.7 and 0.0 Gy, respectively. This test was performed for dynamic delivery to verify that the leaves could adequately shield the central volume, and the TPS modeled the transmission of the leaves correctly. The second test is called a “Step” test, for which the specified dose to each volume was 0.7, 0.5 and 0.3 Gy, respectively. The Step test was for static delivery, to test the delivered accuracy of three relative dose levels. Each institution delineated the predefined volumes for both tests on a local solid water phantom and delivered the specified doses to films (EBT2, International Specialty Products, Wayne, NJ). The reference site centrally evaluated the films from all institutions.

### B. Phantom

**Figure 1:**
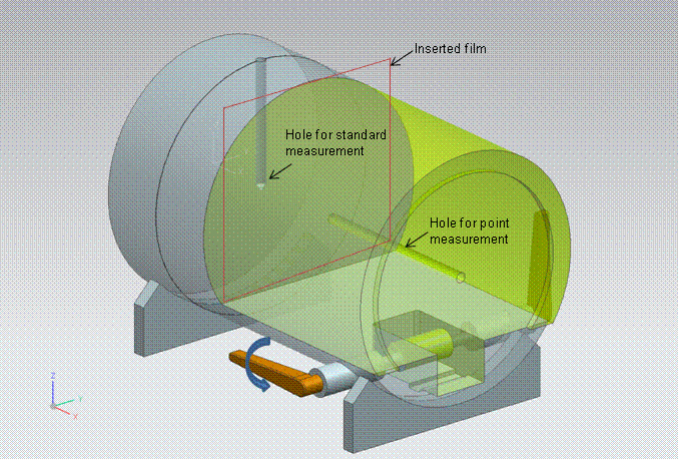
Developed IMRT DQA phantom for LINAC group.

The LINAC group used the same custom‐made phantom of acryl to measure point dose and axial plane dose distributions. The phantom of cylinder, 265 mm in length and 180 mm in diameter, has two holes where an ion chamber can be inserted. It was cut into two pieces, as shown in Fig. 1. The two pieces of phantom (gray and green parts in Fig. 1) were tightened using the lever after inserting a film in order to reduce the air gap between them. One hole at 5 cm depth below the anterior surface was used to measure a conversion factor [nC/cGy] of chamber reading‐to‐dose. This standard measurement was also intended to exclude the daily variation of machine output. The other hole along the axis was used to measure a planned dose in a high‐ or low‐dose and low‐gradient region. The holes were designed especially for a 0.125 cc ion chamber (Semiflex, PTW, Freiburg, Germany). For the TOMO group, the commercial phantom (i.e., ‘cheese’ phantom; (Accuray, Sunnyvale, CA) was used to measure point doses and coronal plane dose distributions. Details of the measurements using this phantom are described elsewhere.^(^ ^13^ ^)^

### C. Mock structures

The AAPM TG‐119 produced the mock structures for the prostate, head and neck (H&N), C‐shape, and multitarget. Each test included the targets, normal structures, planning specifications of dose goals, and beam arrangements. This study followed the general guideline of the AAPM TG‐119 programs. However, the target volumes and OAR (organs at risk) locations of the mock prostate and H&N were based on Korean patients' anatomy.^(^ ^14^ ^)^ The rest of the AAPM TG‐119 mock structures were identically applied for this study. All mock structures were segmented by one physicist from the reference site on DICOM CT images (DICOM, Rosslyn, VA) of both phantoms. Then these DICOM files were centrally distributed to all the institutions to eliminate any institutional variations during segmentation. It was recommended that a grid size of dose calculation used be less than 3 mm and that the calculation algorithm used be the convolution–superposition or equivalent for inhomogeneity correction.^(^ ^15^ ^,^ ^16^ ^)^

#### C.1 Structures

**Figure 2:**
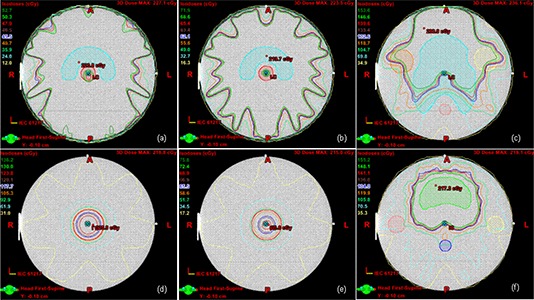
Isodose lines for low‐dose point measurement with mock structures: (a) C‐shape easy, (b) C‐shape hard, (c) H&N, (d) superior of multitarget, (e) inferior of multitarget, and (f) prostate.

For the prostate test, the mean PTV (planning target volume) and CTV (clinical target volume) volumes of sample Korean patients were 141 cc and 50 cc, respectively. The PTV was defined to include a 1.0 cm margin around the prostate in all directions, except the posterior direction where a 0.5 cm margin was added. The rectum was a cylinder with a diameter of 1.5 cm (mean volume 10 cc) and the bladder was a semi‐ellipsoidal shape (mean volume 144 cc). The PTV included about one‐third of the rectum and bladder volumes. Unlike AAPM TG‐119, the femoral heads of spherical shape were added in this test suite. For the H&N test, the mean PTV volume of sample Korean patients was 534 cc. PTV included all anterior volume from the base of the skull to the upper neck and the posterior neck node. Both parotids were delineated with two “truncated cones” (i.e., a cone with the top cut off) of 1.5 cm diameter of a circular top and 2.4 cm diameter of a circular bottom opposing the circular bottom with 30 cc volume and located at the superior aspect of the PTV. The cord was a cylinder shape with a diameter of 1.5 cm. A gap between the cord and PTV was about 1.3 cm. For the C‐shape and multitarget tests, the AAPM TG‐119 structures were used as is. The mock structures are shown in Fig. 2.

#### C.2 Beam arrangements

For the prostate and multitarget tests, the plan had seven fields at 50° intervals from the vertical (e.g., 0°, 50°, 100°, 150°, 210°, 260°, and 310°). For H&N and C‐shape tests, the plan had nine fields at 40° intervals from the vertical (e.g., 0°, 40°, 80°, 120°, 160°, 200°, 240°, 280°, and 320°).

#### C.3 Dose goals

The total prescription dose was 80 Gy (daily dose of 2Gy×40fx) for the prostate test. The total prescription dose of 50 Gy (daily dose of 2Gy×25fx) was applied for H&N, C‐shape, and multitarget tests. The plan goals of C‐shape were divided into easy and hard versions. The specific planning goals are shown in Table 2.

**Table 2:** Treatment plan goals and results for all tests of both groups.

*Test*	*Group*			*LINAC*			*TOMO*	
	*Parameter*	*Goal*	*Mean*	*STDV*	*COV*	*Mean*	*STDV*	*COV*
Prostate	PTV D95	>7600	7620.4	52.9	0.007	7788.3	166.3	0.021
	PTV D5	<8400	8267.4	188.1	0.023	8111.0	118.3	0.015
	Rtm D30	<7000	6630.6	392.4	0.059	6270.0	628.6	0.100
	Rtm D10	<7500	7324.6	208.7	0.028	7694.0	146.9	0.019
	Bld D30	<7000	5452.7	738.7	0.135	5346.7	660.3	0.124
	Bld D10	<7500	7414.8	144.8	0.020	7729.3	233.2	0.030
	RtF D10	<5000	4127.4	503.4	0.122	4171.7	926.4	0.222
	LtF D10	<5000	4014.8	504.5	0.126	4112.7	768.7	0.187
H&N	PTV D90	5000	5052.9	84.6	0.017	4996.7	70.2	0.014
	PTV D99	>4650	4784.1	94.8	0.020	4883.3	98.7	0.020
	PTV D20	<5500	5289.6	147.2	0.028	5215.7	149.3	0.029
	Cd max	<4000	3915.0	257.5	0.066	3282.0	499.2	0.152
	RtPd D50	<2000	1916.7	160.2	0.084	1438.3	192.4	0.134
	LtPd D50	<2000	1887.3	135.3	0.072	1394.3	144.7	0.104
C‐shape(E)	PTV D95	5000	4985.6	64.8	0.013	4982.0	19.3	0.004
	PTV D10	<5500	5463.3	188.6	0.035	5437.7	207.7	0.038
	Core D10	<2500	2446.3	145.1	0.059	1793.3	583.2	0.325
C‐shape(H)	PTV D95	5000	4937.0	116.6	0.024	4790.7	116.8	0.024
	PTV D10	<5500	5639.4	162.7	0.029	5950.0	578.2	0.097
	Core D10	<1000	1552.9	211.9	0.136	1178.3	361.4	0.307
Multiple‐target	Ct D99	>5000	4975.6	54.0	0.011	4918.0	74.6	0.015
	Ct D13	<5300	5417.2	117.1	0.022	5852.7	1002.6	0.171
	Sup D99	>2500	2676.2	204.2	0.076	2437.3	84.5	0.035
	Sup D13	<3500	3521.3	352.0	0.100	3700.7	149.9	0.041
	Inf D99	>1250	1430.3	353.2	0.247	1220.0	60.8	0.050
	Inf D13	<2500	2593.5	607.2	0.234	3262.7	423.8	0.130

PTV=planning target volume; COV=coefficient of variation; STDV=standard deviation; Rtm=rectum; Bld=bladder; RtF=right femoral head; LtF=left femoral head; Cd=cord; RtPd=right parotid; LtPd=left parotid; Ct=central target; Sup=superior target; Inf=inferior target.

#### C.4 Measurement location

**Figure 3:**
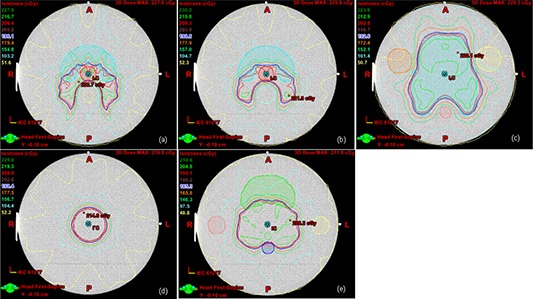
Isodose lines for high‐dose point measurements and composite field plane with mock structures: (a) C‐shape easy, (b) C‐shape hard, (c) H&N, (d) multitarget center, and (e) prostate.

A point for high‐dose measurements was always the isocenter in the middle of PTV, where doses were high and uniform (see Fig. 3). A point for low‐dose measurements was located in the OAR structure such as 3 cm posterior to the rectum for the prostate, 4 cm posterior in midspinal cord for H&N, the center of cord for the C‐shape, and the center of either of two outer targets for the multitarget (see Fig. 2). For the per‐field measurements, a plane perpendicular to the beam axis was located at 5 cm depth in a water‐equivalent phantom with the SAD (source‐to‐axis distance) setup. The per‐field measurement was only performed for H&N test and limited to the LINAC group. A plane for the composite field measurement was across the isocenter. The plane selected for the composite field measurement often included the points for high‐ and low‐dose measurements.

### D. Point measurements

The LINAC group used the phantom of Fig. 1 using a 0.125 cc ion chamber (Semiflex, PTW, Freiburg, Germany). Two sets of phantom CT images (i.e., vertical and horizontal positions) were acquired with a slice thickness of 2.5 mm. The vertical phantom images were used to calculate MUs in TPS for a conversion factor, where it was assumed that the vertical phantom at 100 cm SSD be irradiated by a $10\times 10\;{\text{cm}}^{2}$ field (so‐called “standard measurements”). The horizontal phantom images were used to calculate point dose values for all tests by assuming the horizontal phantom was irradiated by all fields as planned in TPS. These planned values were compared with the measured doses. In the TOMO group, the cheese phantom was used to measure a point dose using a 0.05 cc ion chamber (Exradin A1SL, Standard Imaging Inc., Middleton, WI). The TOMO group measured an absolute dose for a standard measurement using $N_{D,W}^{C0-60}$ and $k_{Q}$ values. The rest of the procedure for comparison was the same as those in the LINAC group. In the low‐dose measurement, the C‐shape plan had the lowest dose in the OAR, which was expected to be at least 30 cGy. Therefore, the issue regarding very low‐dose measurements would not arise in this study.^(^ ^8^ ^)^

### E. Planar dose measurements

The per‐field measurements were done by the LINAC group only for the H&N test. Each institution in the LINAC group used either a detector array or film; two institutions used MapCHECK (Sun Nuclear Corporation, Melbourne, FL); one institution used the ion chamber array (PTW, Freiburg, Germany); two institutions used MatriXX (IBA Dosimetry GmbH, Schwarzenbruck, Germany); and two institutions used EBT2 films (International Specialty Products, Wayne, NJ). The individual fields were delivered at gantry 0°. This avoided the angular dependency of detectors during per‐field measurements.^(^ ^17^ ^,^ ^18^ ^)^ All institutions were asked to perform the composite field measurements using films and the custom‐made (LINAC) or cheese (TOMO) phantom. The multitarget test was excluded from this measurement. The films for composite field measurement were evaluated using two gamma criteria of 2 mm DTA/2% dose difference and 3 mm DTA/3% dose difference. The planar dose distributions were normalized at a reference (dose to the isocenter) or maximum dose in a low‐gradient region. The region of interest (ROI) was first specified as a maximum size of rectangle on the film. Then any pixels that received less than 10% of the maximum dose in the dose map were excluded from the gamma evaluation. Thus, the excluded points were outside of ROI.

## III. RESULTS

### A. Output and TPS audit

The results of output audit for all participating institutions ranged from −1.8% to +2.4%, which indicated that all of them passed the study criteria of less than 3%. Six institutions undertook Dip and Step tests for the TPS audit, and all test plans met the required dose constraints. Four of the institutions did not perform these tests, but were requested to present the results of local TPS commission tests with film measurements. The average percentage of points passing the gamma criteria of 3 mm/3% in the Dip and Step tests were 98.2% (97.4% to 99.2%) and 97.8% (97.2% to 98.6%), respectively.

### B. Planning results

The statistics of the mock plans for both groups are presented in Table 2. In this table, Dxx means the dose covering xx% of the volume. The coefficient of variation (COV) was a normalized measurement of the dispersion of a probability distribution that was defined as a ratio of the standard deviation (STDV) to the mean.

### C. Point dose measurements

In this study, the dose difference was expressed as a ratio of the difference between measured and planned doses to the planned dose, instead of the prescription dose used in AAPM TG‐119. The dose difference is planned according to the following equation:

(1) $$\text{Dose}\;\text{difference}(\%)=\frac{\text{Measured}\;\text{dose}-\text{Planned}\;\text{dose}}{\text{Planned}\;\text{dose}}\times 100(\%)$$

The planned dose from TPS was a value at a point of measurement for comparison. In high‐dose measurements, the average difference between measured and planned doses averaged over all tests was −0.7%±1.2% for LINAC, −0.5%±1.4% for TOMO, and −0.6%±1.3% for all institutions. It ranged from −3.3% to 1.9% for the LINAC group and from −2.5% to 2.9% for the TOMO group. The maximum dose difference occurred in the hard C‐shape structure for both groups. The average confidence limit was 3.1% for both groups. The results of these measurements for both groups are summarized in Table 3.

**Table 3:** Results of high‐dose point measurement, averaged over the institutions for LINAC/TOMO/Total groups.

*Test*	*Mean (%)*	*STDV(%)*	*CL(%)*	*No.*
Multitarget	−0.3/−0.2/−0.2	1.0/1.8/1.2	2.3/3.6/2.5	7/3/10
Prostate	−0.0/−0.6/−0.3	1.0/1.2/1.1	1.9/3.0/2.4	7/6/13
H&N	−1.0/−1.3/−1.1	0.7/0.8/0.7	2.4/2.8/2.6	7/6/13
C‐shape(E)	−1.3/0.2/−0.9	1.1/1.4/1.4	3.5/3.0/3.5	7/3/10
C‐shape(H)	−0.8/0.5/−0.4	1.9/2.2/2.0	4.5/4.7/4.2	7/3/10
Overall combined	−0.7/−0.5/−0.6	1.2/1.4/1.3	3.1/3.2/3.1	35/21/56

E=easy; H=hard; and CL=confidence limit.

During the high‐dose measurement analysis, two institutions showed abnormal values larger than ±5%. Both institutions were informed of these large discrepancies and they then repeated the measurements. They carefully checked the whole process and found that the conversion factor from the standard measurement was incorrect. After the correction, the results from both institutions were then less than 3%. In low‐dose measurements, the average difference between measured and planned doses averaged over all tests was −1.0%±1.9% for LINAC group, 0.1%±2.5% for TOMO group, and −0.6%±2.2% for all institutions. It ranged from −6.0% to 3.5% for the LINAC group and from −3.4% to 6.6% for TOMO group. The maximum dose difference also occurred in the hard C‐shape structure for both groups. The average confidence limit was 4.9% for both groups. The results of these measurements are shown in Table 4. The local confidence limit of each institution, averaged over all the test plans, is listed in Table 5.

**Table 4:** Results of low‐dose point measurement, averaged over the institutions for LINAC/TOMO/Total groups.

*Test*	*Mean (%)*	*STDV(%)*	*CL(%)*	*No.*
MT (suf)	−0.3/1.0/0.1	1.8/2.7/2.1	3.9/6.3/4.1	7/3/10
MT (inf)	−0.5/−0.1/−0.4	1.6/3.0/1.9	3.3/6.0/4.0	7/3/10
Prostate	−1.5/−1.0/−1.2	1.3/1.2/1.2	3.9/3.4/3.6	7/6/13
H&N	−1.7/−0.5/−1.1	0.8/2.1/1.6	3.3/4.5/4.2	7/6/13
C‐shape(E)	0.2/−0.7/−0.1	2.3/2.6/2.3	4.8/5.7/4.6	7/3/10
C‐shape(H)	−2.3/3.7/−0.5	2.6/2.6/3.8	7.4/8.7/7.9	7/3/10
Overall	−1.0/0.1/−0.6	1.9/2.5/2.2	4.8/5.0/4.9	42/24/66

MT=multitarget; E=easy; H=hard; and CL=confidence limit.

**Table 5:** Results of dose point measurement, averaged over all the test plans measured at each.

*Measurement*	*Group*				*LINAC*					*TOMO*	
	*Institution*	*A*	*B*	*C*	*D*	*E*	*F*	*G*	*H*	*I*	*J*
High Dose	Mean(%)	−1.0	−1.1	−0.4	0.3	−1.8	−0.3	−0.5	−1.1	−0.1	−0.1
	TDV(%)	0.8	1.6	1.2	0.8	1.4	0.5	1.5	1.3	1.7	1.1
	LCL(%)^a^	2.6	4.3	2.7	1.9	4.5	1.2	3.4	3.6	3.4	2.3
	No.	5	5	5	5	5	5	5	7	7	7
Low Dose	Mean(%)	−1.5	−1.7	0.4	−0.7	−2.2	−1.7	0.4	−0.6	1.1	−0.1
	STDV(%)	1.0	2.4	1.7	1.8	2.0	1.3	1.8	2.1	3.3	1.6
	LCL(%)^a^	3.4	6.4	3.7	4.1	6.2	4.3	4.0	4.7	7.7	3.3
	No.	6	6	6	6	6	6	6	8	8	8

a LCL=local confidence limit

### D. Planar dose measurements

The planar dose distributions were assessed using two gamma criteria of 2 mm/2% and 3 mm/3%. Recently, several studies reported the statistical correlation between planar IMRT QA passing rates and clinical relevance using a DVH‐based method.^(^ ^19^ ^–^ ^22^ ^)^ However, the current study was based on the mock structures and didn't intend to predict any clinical relevance with DQA results. Thus, the method for per‐field and composite field analysis coincided with the AAPM TG‐119 tolerance limit metrics using the concept of confidence limit.

#### D.1 Per‐field measurements

The per‐field measurements were done by seven institutions of the LINAC group only. The H&N test was selected for the per‐field measurements since it had the most complicated dose distributions. Each institution used an available tool such as a detector array or films. The average percentage of points passing the gamma criteria of 2 mm/2% and 3 mm/3% was 92.7%±6.5% and 98.2%±2.8%, respectively. The corresponding confidence limit was 79.1% and 92.7% (see Table 6). The local confidence limit ranged from 71.4% to 95.3% for 2 mm/2% criteria and from 88.3% to 100% for 3 mm/3% criteria (Table 7).

**Table 6:** Per‐field measurement: averaged percentage of points passing gamma criteria of 2 mm/2% and 3 mm/3% over the institutions.

*Criteria*	*Mean(%)*	*STDV(%)*	*No.*	*CL(%)*
2 mm/2%	92.7	6.5	63	79.1
3 mm/3%	98.2	2.8	63	92.7

**Table 7:** Per‐field measurement: local averaged percentage of points passing gamma criteria of 2 mm/2% and 3 mm/3%, with associated confidence limits.

*Criteria*	*Institution*	*A*	*B*	*C*	*D*	*E*	*F*	*G*
	*Device*	*MapCHECK*	*MapCHECK*	*EBT2*	*MatriXX*	*EBT2*	*2D‐Array*	*MatriXX*
2 mm/2%	Mean(%)	95.2	93.0	90.5	96.3	79.1	96.8	98.1
	STDV(%)	2.9	1.7	5.4	1.0	4.0	2.7	1.5
	LCL(%)	89.4	89.7	80.0	94.4	71.4	91.5	95.3
3 mm/3%	Mean(%)	99.4	99.3	98.0	99.0	92.4	99.0	100.0
	STDV(%)	1.0	0.5	2.1	0.9	2.1	1.3	0.0
	LCL(%)	97.4	98.3	93.9	97.2	88.3	96.5	100
	No.	9	9	9	9	9	9	9

#### D.2 Composite film measurements

The composite film measurements for all mock tests, except for the multitarget, were performed by all institutions of both groups. The TOMO group measured one additional set of tests for the prostate and H&N structures. Table 8 summarizes the percentage of points passing the gamma criteria that was averaged over all institutions of both groups and the associated confidence limits. The gamma passing rate averaged over all mock test plans in the LINAC group was 84.7%±7.5% for 2 mm/2% criteria and 94.6%±4.0% for 3 mm/3% criteria. The gamma passing rate averaged over all mock test plans in the TOMO group was 88.4%±3.7% for 2 mm/2% criteria and 96.4%±3.2% for 3 mm/3% criteria. The gamma passing rate averaged over all mock test plans in all institutions was 86.1%±6.5% for 2 mm/2% criteria and 95.3%±3.8% for 3 mm/3% criteria. The TOMO group showed a higher passing rate and a lower standard deviation than the LINAC group. The local confidence limit is summarized in Table 9.

**Table 8:** Composite film: percentage of points passing gamma criteria of 2 mm/2% and 3 mm/3%, with associated confidence limits for LINAC/TOMO/Total groups.

*Criteria*	*Test*	*Prostate*	*H&N*	*C‐shape(E)*	*C‐shape(H)*	*Overall*
2 mm/2%	Mean(%)	86.5/88.1/87.2	85.2/86.6/85.7	85.4/92.1/88.8	81.7/88.6/83.2	84.7/88.4/86.1
	STDV(%)	6.1/2.9/4.8	7.3/0.9/5.7	8.3/7.4/8.2	9.1/3.2/8.5	7.5/3.7/6.5
	CL(%)	74.6/82.4/77.9	70.9/84.8/74.5	69.1/77.6/72.7	63.9/82.4/66.6	69.9/81.1/73.3
3 mm/3%	Mean(%)	95.4/96.4/95.8	94.6/96.1/95.3	95.1/98.0/96.0	93.3/95.5/94.0	94.6/96.4/95.3
	STDV(%)	3.4/3.6/3.4	3.4/4.2/3.7	4.1/1.5/3.7	5.3/2.1/4.6	4.0/3.2/3.8
	CL(%)	88.7/89.3/89.2	87.9/87.8/88.0	87.1/95.1/88.7	82.9/91.3/85.0	86.8/90.0/87.9
	No.	7/6/13	7/6/13	7/3/10	7/3/10	28/18/46

**Table 9:** Composite film: percentage of points passing gamma criteria of 2 mm/2% and 3 mm/3%, averaged over the test plans, with associated confidence limits.

*Criteria*	*Group*				*LINAC*					*TOMO*	
	*Institution*	*A*	*B*	*C*	*D*	*E*	*F*	*G*	*H*	*I*	*J*
2 mm/2%	Mean	91.1	81.0	77.2	84.1	78.9	95.4	85.0	86.3	89.0	89.8
	LCL	82.6	78.5	67.7	70.6	62.6	90.4	83.2	82.0	80.8	81.8
3 mm/3%	Mean	98.0	93.1	90.1	95.4	90.7	99.3	95.7	96.6	93.8	98.8
	STDV	1.3	0.7	3.2	3.4	4.3	0.6	1.2	3.0	2.9	1.7
	LCL	95.5	91.7	83.9	88.7	82.2	98.1	93.4	90.7	88.1	95.5
	No.	4	4	4	4	4	4	4	6	6	6

#### D.3 Correlation between the magnitude of point dose error and gamma passing rates of composite field measurements

The point dose error is an absolute value of dose difference as defined below:

(2) $$\text{Point}\;\text{dose}\;\text{error}(\%)=\left|\frac{\text{measured}\;\text{dose}-\text{Planned}\;\text{dose}}{\text{Planned}\;\text{dose}}\right|\times 100(\%)$$

**Figure 4:**
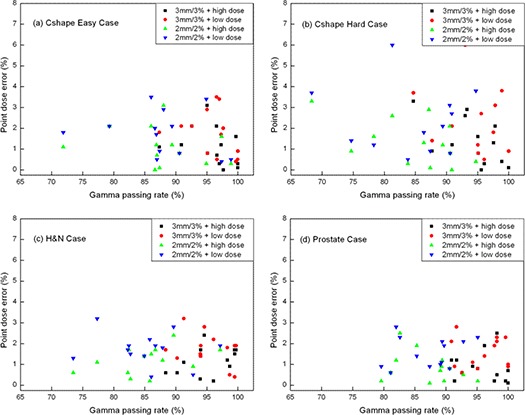
Magnitude of the point dose errors (low dose and high dose) vs. composite filed gamma passing rates (2 mm/2% and 3 mm/3%) for each test: (a) C‐shape easy, (b) C‐shape hard, (c) H&N, and (d) prostate.

Figure 4 shows the magnitude of the point dose errors (low‐ and high‐dose measurements) versus composite field gamma passing rates (2 mm/2% and 3 mm/3%) for each test. The gamma passing rates for 2 mm/2% criteria ranged much broader than those for 3 mm/3%, while there was no significant difference in point dose errors between both criteria. However, data points for C‐shape hard cases were the most broadly dispersed, while data points for prostate case were the most closely confined. There was no stringent correlation between the magnitude of point dose errors and gamma passing rates for composite field measurements in this study.

## IV. DISCUSSION & CONCLUSION

The multi‐institutional joint research was first performed in Korea to suggest tolerance levels for the IMRT DQA measurements. We followed the confidence limit concept presented in the AAPM TG‐119 report. Venselaar et al.^(^ ^23^ ^)^ first suggested that the confidence limit concept was expressed with the mean value and the standard deviation (SD) multiplied by 1.5 to quantify the dose accuracy of photon beam calculations of 3D treatment planning. In AAPM TG‐119 and this study, a confidence probability p=0.05 (confidence limit=|mean+1.96 SD|) was applied. In this study, a concept of confidence limit for the point dose measurements was appropriate metrics because of a small standard deviation and a large number of samples.^(^ ^24^ ^)^ However, for the planar dose gamma evaluation with 2 mm/2% criteria, the concept of confidence limit was not appropriate metrics because the data including the local measurements have not only a large local deviation, but were also from a small number of samples. With 3 mm/3% criteria, it was also difficult to define the confidence limit because of a small number of samples. There was no significant difference in the tolerance levels of point dose measurements between LINAC and TOMO groups. In spite of the differences in mock structures and dosimetry tools, our tolerance levels agreed with those of AAPM and ESTRO guidelines.

The result can be used for a comparison guide of other institutions in Korea as they evaluate their IMRT commissioning and DQA results. In the near future, the domestic audit of IMRT DQA will be carried out by comparing institutional local values with the confidence limits determined by this study.

## ACKNOWLEDGMENTS

This work was in part supported by the National Research Foundation of Korea (800‐20110212 and 490‐20120026) grant funded by the Korea government (MEST).
